# Supplementary material for: Investigation of hs-TnI and sST-2 as Potential Predictors of Long-Term Cardiovascular Risk in Patients with Survived Hospitalization for COVID-19 Pneumonia
Source: Biomedicines. 2022 Nov 10;10(11):2889. doi: 10.3390/biomedicines10112889 (PMC9687975; doi:10.3390/biomedicines10112889)
Supplement: Supplementary file 1 [file biomedicines-10-02889-s001.zip › biomedicines-1911917-supplementary.pdf]

Supplemental tables

Table S1 Applied specific COVID-19 therapies during hospitalization.

Treatment	% (*n*)
JAK-inhibitors	15.9 (40)
Corticosteroids	87.6 (220)
IL-6 antagosnist	75.3 (189)
Therapeutic anticoagulation	74.7 (165)
Remdesivir	100 (251)

JAK-janus kinase, IL-6-interleukin-6

Table S2 Relevant cardiovascular therapies in COVID-19 survivors after hospital discharge

Treatment	% (*n*)
Statins	6.0 (15)
Betablockers	11.1 (28)
ACE-blockers /Angiotensin receptor anatogonists	20.7 (52)
Cardioselective caliuman channel blockers	1.2 (3)
Diuretics	3.6 (9)
Acetylsalicylic acid	8.0 (20)
Clopidogrel	1.6 (4)
Aldosteron anatgonists	4.0 (10)
Ivabradine	0.4 (1)

ACE-aniotensin converting enzyme

Table S3 Univariate Analysis for prediction of MACE during follow-up after hospital discharge in COVID-19 survivors (cut off<0.05)

	coef±SE	HR	95% CI	*p*
Age	0.0493±0.0188	1.0506	1.012 – 1.090	**0.009****
Gender	0.5838±0.4495	1.7928	0.743 – 4.327	0.194
BMI	0.0106±0.0435	1.0107	0.928 – 1.101	0.806
SpO_2, %	0.1523±0.1245	1.1646	0.912 – 1.486	0.221
Temperature at admission, °C	0.3109±0.2749	1.3646	0.796 – 2.339	0.258
SAP	0.0246 ±0.0129	1.0249	0.999 – 1.051	0.056
DAP	0.0453 ±0.0241	1.0463	0.998 – 1.097	0.061
BR	-0.1213 ±0.2158	0.8858	0.580– 1.352	0.574
Lung tissue damage on CT	-0.0006 ±0.0015	0.9994	0.997– 1.002	0.702
AH	0.7322±0.4495	2.0797	0.862 – 5.019	0.103
DM	-0.2878±1.0260	0.7499	0.101 – 5.602	0.779
CKD	-16.02±596.5	0.0000001	–	0.998
CHD	-17.08±518.8	0.00000004	–	0.997
CHF	-16.03±463.2	0.0000001	–	0.997
COPD+BA	-0.6069±1.0260	0.5450	0.073 – 4.071	0.554
Procalcitonin	1.533±2.382	4.632	0.043 – 493.7	0.520
Hb	0.0209±0.0152	1.0211	0.991 – 1.052	0.170
WBC	0.0007±0.0647	1.0007	0.882 – 1.136	0.991
Platelets	0.0039±0.0029	1.0039	0.998 – 1.010	0.169
CRP	0.0104±0.0093	1.0104	0.992 – 1.029	0.268
Albumin	0.0698±0.0666	1.0723	0.942 – 1.221	0.292
GFR	0.0124±0.0128	1.0125	0.987 – 1.038	0.334
Sodium	-0.0306 ±0.0699	0.9699	0.846 – 1.112	0.662
Potassium	-0.0522 ±0.1596	0.9491	0.694 – 1.298	0.743
sST-2	2.617±0.471	13.695	5.441 – 34.477	**<0.001*****
Hs-TnI	1.2195±0.4689	3.3854	1.351 – 8.487	**0.009****

AH–arterial hypertension, BA–bronchial asthma, CK–creatine kinase, CHD–coronary heart disease, CHF-congestive heart failure, CKD–chronic kidney disease, CRP- C-reactive protein, CT computer tomography, CV– cardiovascular, DAP–diastolic arterial blood pressure, DM–Diabetes Mellitus type 2, FU-follow-up, Hb–hemoglobin, MACE-major adverse cardiovascular event, SAP–systolic arterial blood pressure, WBC–white blood count, sST-2-solutable suppression of tumorigenicity 2, hs-TnI-high sensitive Troponin I; bold print=p<0.05, **, ***–significance in p < 0.01, p < 0.001.

Table S4 Multivariate analysis for prediction of MACE during follow-up after hospital discharge in COVID-19 survivors (cut off<0.05)

	Coef ±SE	exp(coef)	CI 95% exp(coef)	p-level
Age	0.046 ±0.021	1.047	1.005 – 1.092	**0.0288***
Gender	0.475 ±0.636	1.608	0.462 – 5.595	0.4550
SBP	0.029 ± 0.019	1.029	0.991 – 1.069	0.1384
DBP	0.018 ±0.034	1.018	0.952 – 1.088	0.6074
HR	0.023 ±0.019	1.023	0.986 – 1.063	0.2258
Hb	0.001 ±0.017	1.001	0.967 – 1.036	0.9476
GFR	0.022 ±0.019	1.022	0.985 – 1.060	0.2455
ST2	0.002 ±0.001	1.002	1.0005 – 1.004	**0.0215***
Troponin I	0.297 ±0.124	1.345	1.056 – 1.716	**0.0169***
Concordance= 0.777	Likelihood ratio test= 28.23, p<0.001	Wald test = 28.89, p<0.001	Score (logrank) test = 44.4, p<0.001	

*–significance in p < 0.05.
